# Distribution, Fraction, and Ecological Assessment of Heavy Metals in Sediment-Plant System in Mangrove Forest, South China Sea

**DOI:** 10.1371/journal.pone.0147308

**Published:** 2016-01-22

**Authors:** Ruili Li, Minwei Chai, Guo Yu Qiu

**Affiliations:** 1 Shenzhen Key Laboratory for Heavy Metal Pollution Control and Reutilization, School of Environment and Energy, Shenzhen Graduate School of Peking University, Shenzhen, 518055, China; 2 Shenzhen Key Laboratory of Environment Simulation and Pollution Control, PKU-HKUST Shenzhen-HongKong Institute, Shenzhen, 518057, PR China; 3 School of Environment and Energy, Shenzhen Graduate School of Peking University, Shenzhen, 518055, China; Sun Yat-Sen University, CHINA

## Abstract

Overlying water, sediment, rhizosphere sediment and mangrove seedlings in the Futian mangrove forest were analyzed for heavy metals. The results showed that mangrove plant acidified sediment and increased organic matter contents. Except for chromium (Cr), nickel (Ni) and copper (Cu) in *Aegiceras corniculatum* sediment, heavy metals in all sediments were higher than in overlying water, rhizosphere sediment and mangrove root. Heavy metals in *Avicennia marina* sediments were higher than other sediments. The lower heavy metal biological concentration factors (BCFs) and translocation factors (TFs) indicated that mangrove plant adopted exclusion strategy. The geo-accumulation index, potential ecological risk index and risk assessment code (RAC) demonstrated that heavy metals have posed a considerable ecological risk, especially for cadmium (Cd). Heavy metals (Cr, Ni, Cu and Cd) mainly existed in the reducible fractions. These findings provide actual heavy metal accumulations in sediment-plant ecosystems in mangrove forest, being important in designing the long-term management and conservation policies for managers of mangrove forest.

## Introduction

Heavy metal contaminants have gained attention increasingly since recent decades due to their persistence and biotoxicity in the water and soil environment [1–3]. Generally, the contaminants are released into ambient environments mainly via raw or insufficiently treated industrial wastewater effluents, vehicle emissions, mining and the combustion of coals [4, 5]. Located in estuaries or along coastlines, mangrove ecosystem contributes to metal pollution remediation by redistributing pollutants between the land and sea in their biogeochemical cycles. Generally, mangrove increases metal accumulation in sediments by modifying the soil acidity, redox potential, organic contents and salinity [6, 7], and subsequently reduces metal exposure to adjacent aquatic environment [8]. However, it is probable for metals being released and transported from sediments to water when soil physicochemical properties change, which may change their chemical speciation and cause potential ecological impacts and human health issues [9–12].

The systematical quantitative evaluation of heavy metal contamination contributes to the understanding of the potential ecological risk [13]. The most commonly cited assessment indices in environmental studies include geo-accumulation index (*I*_*geo*_), potential ecological risk index (RI), risk assessment code (RAC), enrichment factors (EF) and mean probable effect level quotient (m-P-Q). These methods are widely used to evaluate the heavy metal pollution in farming soils [14], urban soils [15], mine soils [16], and lake sediments [17]. In order to update the comprehensive understanding on this problem, the integrated application of multi-assessment methods to evaluate the ecological risk is important [18–21].

Futian mangrove forest is the only mangrove located in the middle of Shenzhen, South China (adjacent to Mai Po Nature Reserve, Hong Kong) and has suffered serious heavy metal pollution since the early 1990s [22, 23]. Previous studies mainly focused on heavy metal accumulation in mangrove plants and sediments [22–25]. Up to now, no systematic and specialized research has focused on heavy metals in sediment-mangrove plant systems, which are important for understanding heavy metal geochemical cycling in mangrove ecosystem. Based on the discussion above, it was hypothesized that sediment accumulated most of the heavy metals in the whole mangrove system, thereby alleviating their toxicity. Consequently, this study was conducted to (1) determine the occurrence and distribution of heavy metals (Cr, Ni, Cu, As and Cd) in overlying water, sediments, rhizosphere sediments and mangrove seedlings; (2) evaluate heavy metal contamination degrees and potential ecological risk in sediment; (3) investigate the speciation of heavy metals in sediment.

## Materials and Methods

### 2.1 Study area

**Fig 1:**
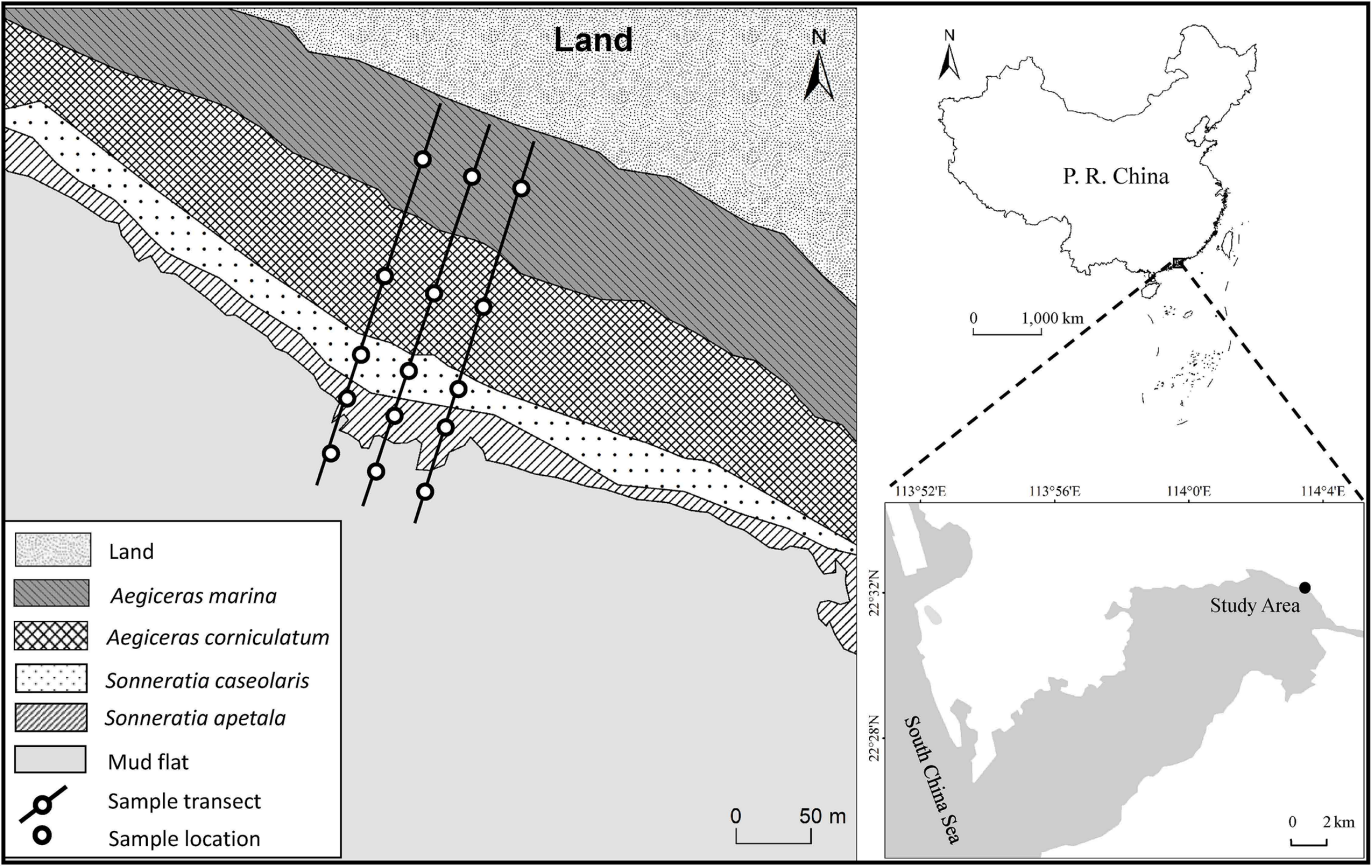
The location of study area.

This study was carried out in the Futian National Nature Reserve (22°31.56′N, 114°00.40′E), located in the northeast coast (Fig 1) of Shenzhen Bay, with a length of 9 km along the coastline and a total area of 368 hectares. The Futian Nature Reserve is the smallest national reserve in China and the only one located in the hinterland of the modern metropolis, only 2.2 km away from the center of Shenzhen city. The mean annual temperature is 22.4°C and the mean annual precipitation is 1700–1900 mm mainly in April to September. The tides in Shenzhen Bay are semi-diurnal, with an average range of 1.9 m.

### 2.2 Sample preparation

In November 2012, sediment, rhizosphere sediment, overlying water and seedlings samples were collected (Permit obtained from Neilingding-Futian National Nature Reserve of Guangdong, Futian National Nature Reserve; No specific permissions were required for the sample location, due to only some sediment, overlying water and mangrove seedlings were sampled; The land is protected and no endangered or protected species were sampled.). From land to sea, four mangrove species were distributed as follows (Fig 1): *Avicennia marina* (native) and *Aegiceras corniculatum* (native), *Sonneratia caseolaris* (introduced) and *Sonneratia apetala* (introduced). An adjacent unvegetated mud flat zone was chosen as a control (5 m from the forest edge), which had similar hydrological conditions and dissimilar biogeochemical cycles with mangroves [26–29]. Fifteen surficial sediment samples (0–20 cm, three duplicates at each site) were collected using acid-washed PVC pipes of length 80 cm and internal diameter 7.5 cm. After the holes were filled with water, fifteen overlying water samples were taken into acid-washed plastic jars. Twelve seedling samples (three duplicates for each species) with similar size and age were collected. The rhizosphere sediments, 5 mm-thick soil attached to the root surface, were gathered carefully with a plastic scraper. Seedling tissues (leaves, stems and roots) were oven-dried at 80°C until a constant weight was reached. After plants roots and stones were picked out from sediments and rhizosphere sediments, the sediment samples were also oven-dried at 80°C until a constant weight was reached. Then, the dry samples were ground into powder and passed through a 0.5-mm sieve. The remained sample on the sieve mainly contained little plants roots and stones, which were abandoned. The overlying water samples were filtered using 0.45μm filters and stored at 4°C for further analysis.

### 2.3 Determinations

As for sediments and rhizosphere sediments, pH and conductivity were determined in deionized water extracts using a mass ratio of 1:5 (sediment to water). TOC in overlying water and sediment was determined by Analytikjena multi N/C 3100 (Germany). The total heavy metal concentrations were determined by subjecting the sediment samples to microwave digestion in a mixture of 9 ml of nitric acid (HNO_3_), 3 ml of hydrofluoric acid (HF) and 1 ml of hydrochloric acid (HCl). The four fractions of heavy metals were extracted using the improved BCR three step sequential extraction procedure proposed by Rauret et al. (1999) [30]. The four fractions were defined as water/acid soluble fraction (F_1_), reducible fraction (F_2_), oxidizable fraction (F_3_) and residual fraction (F_4_). Plant samples were subjected to microwave digestion in 9 ml HNO_3_ and 1 ml HCl [31, 32]. The total heavy metal concentrations in the whole plant-sediment system, and those in all extracted fractions in sediments were analyzed using inductively coupled plasma mass spectrometry (ICP-MS, Agilent 7500).

The bioaccumulation indices were calculated using biological concentration factor (BCF) and translocation factor (TF) following the methods of Yoon et al. (2006) and Cui et al. (2007) [33, 34].

BCF = concentration in roots / concentration in sediments

TF = concentration in leaves / concentration in roots

### 2.4 Risk assessment methods

Geo-accumulation index (*I*_*geo*_) was originally proposed (Müller 1969) to measure the temporal variation of heavy metals by comparing the present-day metal concentrations in aquatic sediments with the geochemical background (pre-civilized background values) [35]. *I*_*geo*_ is defined as: *I*_*geo*_ = log_2_ *C*_*n*_/1.5*B*_*n*_ where C_n_ is the concentration of metal n measured in this study and B_n_ is the geochemical background value in the upper crust [36, 37]. The constant 1.5 was used to account for possible variation in background value due to lithogenic effect. Seven classes of *I*_*geo*_ were proposed [38]: *I*_*geo*_≤0, uncontaminated (UC); 0<*I*_*geo*_<1, uncontaminated to moderately contaminated (UMC); 1<*I*_*geo*_<2, moderately contaminated (MC); 2<*I*_*geo*_<3, moderately to heavily contaminated (MHC); 3<*I*_*geo*_<4, heavily contaminated (HC); 4<*I*_*geo*_<5, heavily to extremely contaminated (HEC); 5<*I*_*geo*_, extremely contaminated (EC)

The potential ecological risk coefficient ($E_{\text{r}}^{i}$) and potential ecological risk index (RI) were calculated using the following formula [39]:

$$E_{\text{r}}^{i}=T_{\text{r}}^{i}•C_{\text{r}}^{i}=T_{\text{r}}^{i}•C_{s}^{i}/C_{n}^{i}$$

where $C_{\text{f}}^{i}$-the contamination factor, $C_{\text{s}}^{i}$-the concentration of heavy metals in the sediment, and $C_{\text{n}}^{i}$—a reference value for heavy metals. $T_{r}^{i}$-the metal toxic response factor. According to Hakanson (1980), the $T_{r}^{i}$ values for measured metal elements are as follows: Cr = 2, Ni = 5, Cu = 5, As = 10, Cd = 30. The degree of $E_{\text{r}}^{i}$ can be categorized as follows: $E_{\text{r}}^{i}$ < 40: low risk (LR), 40 ≤ $E_{\text{r}}^{i}$ < 80: moderate risk (MR), 80 ≤ $E_{\text{r}}^{i}$ < 160: considerable risk (CR), 160 ≤ $E_{\text{r}}^{i}$ < 320: high risk (HR) and $E_{\text{r}}^{i}$ ≥ 320: very high risk (VHR). RI was classified into four levels: RI < 150: low risk (LR), 150 ≤ RI < 300: moderate risk (MR), 300 ≤ RI < 600: considerable risk (CR) and RI ≥ 600: very high risk (VHR).

The risk assessment code (RAC) was first proposed by Perin et al. (1985) [40] and has been widely applied [2, 10, 41] to evaluate heavy metal pollution in sediments by applying a scale to the percentage of metals present in fraction F1 (water/acid-soluble fraction). A five-level risk classification has been categorized in terms of RAC: no risk (< 1%, NR), low risk (1%–10%, LR), medium risk (11%–30%, MR), high risk (31%–50%, HR), and very high risk (> 50%, VHR).

## Results and Discussion

### 3.1 Physicochemical characteristics

There were no significant change of conductivity among four mangrove species and mud flat (Table 1). pH showed a descending trend from overlying water (pH 7.98–8.25) to rhizosphere sediments (pH 6.85–7.18) and sediments (pH 6.55–7.06) in all the five study zones. These results suggested that mangrove trees may lead to acidification of sediment, which is in agreement with the results of previous studies of mangroves [6, 42, 43] and other marine plants such as *Spartina alterniflora* [44, 45]. Except for *A*. *corniculatum*, the TOC levels decreased towards mud flat in sediment, implying that the sediments inside mangrove forest could retain organic matter. Additionally, the litter decay released nutrients into sediments, which contributes to the increase of organic matter in sediments (Table 1). Overall, mangrove plant improved the sediment fertility and promoted the physicochemical properties of the intertidal habitat, which would influence the biogeochemical processes of other elements (including heavy metals) in the sediment.

**Table 1:** The selected physicochemical properties in overlying water, sediments and rhizosphere sediment from four species and mud flat sites in the Futian mangrove forest, South China Sea.

	From land to Seawater				
	*A*. *marine*	*A*. *corniculatum*	*S*. *caseolaris*	*S*. *apetala*	Mud flat
**Overlying water**					
pH	8.21	7.98	8.14	8.12	8.25
Conductivity (S·m^-1^)	1.66	1.44	1.75	1.73	1.64
TOC (mg·L^-1^)	7.95	7.80	9.70	6.82	6.15
**Sediment**					
pH	6.55	6.86	6.93	6.43	7.06
Conductivity (S·m^-1^)	0.51	0.33	0.40	0.50	0.36
TOC (mg·kg^-1^)	530.50	252.50	485.00	451.00	424.00
**Rhizosphere sediment**					
pH	6.95	7.18	6.85	6.81	
Conductivity (S·m^-1^)	0.70	0.46	0.64	0.53	
TOC (mg·kg^-1^)	288.00	388.00	467.50	462.50	

### 3.2 Heavy metals distributions in overlying water-sediment-mangrove plant root

**Fig 2:**
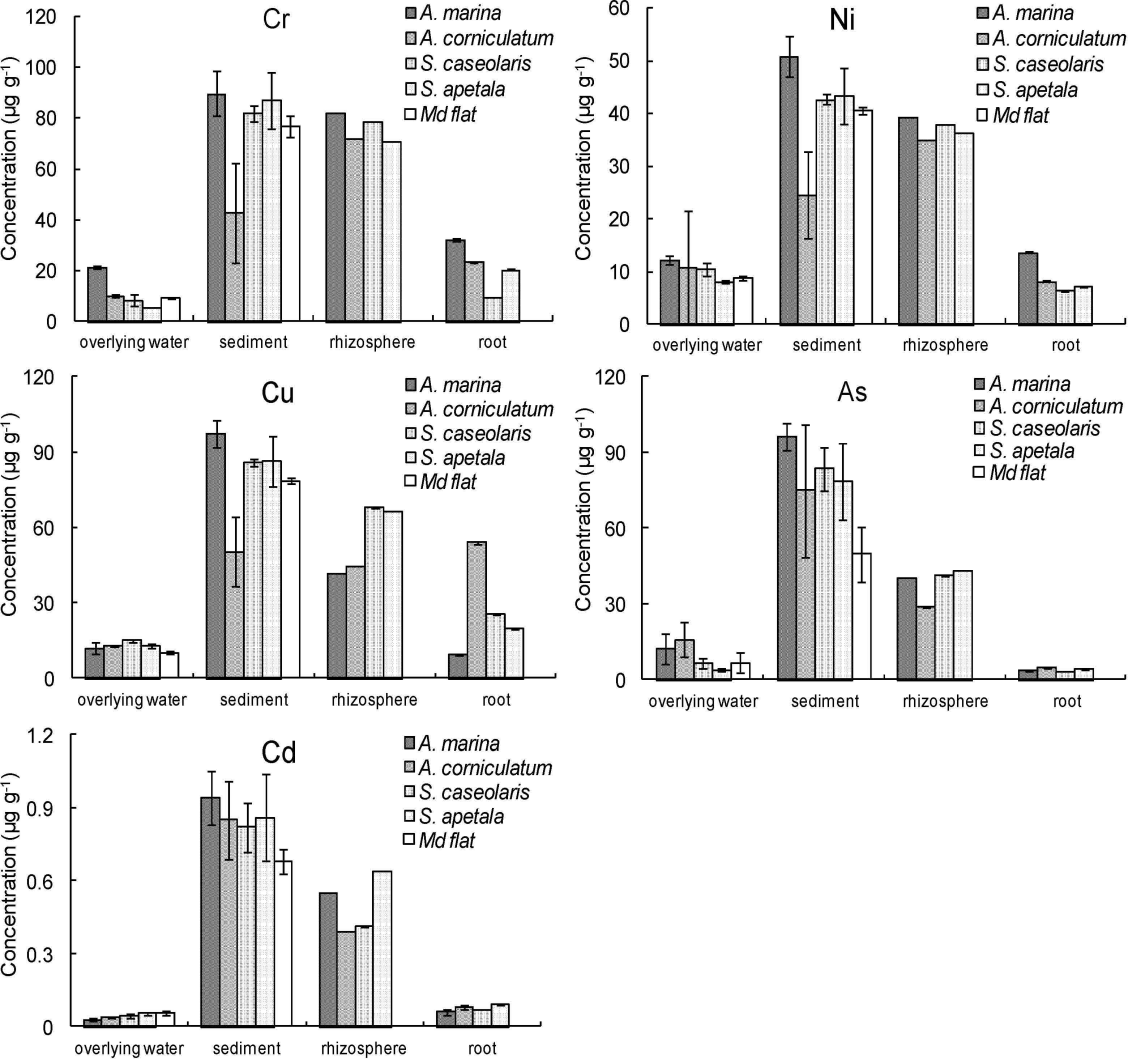
The distributions of heavy metals in overlying water-sediment-rhizosphere soil-root system in the Futian mangrove forest, South China. The sample sites from land to seawater direction are: *A*. *marina*—*A*.*corniculatum*—*S*. *caseolaris*—*S*. *apetala*—Mud flat.

In the present study, the levels of Cr, Ni, Cu and Cd in all sampling sites basically followed the order: sediment > rhizosphere sediment > root > overlying water; while, the distribution of As was sediment > rhizosphere sediment > overlying water > root (Fig 2). The distribution of As in overlying water was higher than root, indicated that As (a kind of metalloid) was not as active as other metals, and could not be absorbed by mangrove root actively as that of other metals. Previously studies have shown that phosphorus competed with arsenic (As) in transfer and accumulation of As in plant [46, 47]. Thus, the large amount of phosphorus deposited in Futian mangrove sediment [48, 49] may compete with As transfer and absorption in mangrove plants, leading to lower As level in mangrove roots. The heavy metal contents in sediments were nearly four times higher than that in overlying water, and four to ten times higher than in mangrove root. Compared to sediment, the lower metal contents in rhizosphere sediment may be related to that mangrove plants passively took up heavy metals from the surrounding sediment [50, 51]. The heavy metals levels were highest in *A*. *marina* sediment, with the lowest levels of Cr, Ni and Cu occurred in *A*. *corniculatum* sediment. The levels of Cr, Ni and Cu in *A*. *corniculatum* zone were lower than other mangrove species and mud flat zone, very well be due to low level of TOC (Table 1), which could be explained, at least partly, by that TOC could regulate metal association in the sediments [52, 53]. The decomposition of organic matter may lead to acidification of sediment. However, no corresponding increased pH was detected for *A*. *corniculatum* (Table 1). In fact, other factors, such as particle size distributions, may also affect heavy metal accumulation [54, 55, 56]. Except for *A*. *corniculatum*, the basic distribution of heavy metal concentrations declined offshore, which may be related to that the landward area was close to the land-sourced pollutant emission outlets. Another possible reason may be due to that the distribution of *A*. *marina* was far away from seawater, and was less affected by tide dilution compared to other mangrove sediments and mud flat.

Generally, biological concentration factors (BCFs) and translocation factors (TFs) are widely used to estimate a plant’s abilities in accumulating metals from soils and transferring metals from roots to shoots, respectively [33]. If plants exhibit BCFs or TFs more than one, they are suitable for phytoremediation [57]. In the present study, BCFs and TFs were basically less than one (except for BCF of Cu in *A*. *corniculatum*, TF of Cu in *A*. *marina* and TF of Cd in *A*.*corniculatum*, Table 2). These results indicated that these mangrove species tend to restrict metal soil-root and root-shoot transformations, guaranteeing the conduction of various important metabolic activities including photosynthesis in the aboveground parts. Among the four mangrove species, *A*. *corniculatum* had the highest BCFs for all heavy metals measured except for Cd. *A*. *marina* and *A*. *corniculatum* had higher BCFs than *S*. *caseolaris* and *S*. *apetala*, which may be related to their location closer to the land, as well as their greater ability in absorbing metals from soils. In Table 2, higher TFs occurred in *Sonneratia* species (except for Cu and Cd), implying their higher sensitive to heavy metals stresses.

**Table 2:** The biological concentration factors (BCFs) and translocation factors (TFs) of heavy metals in selected mangrove species in Futian mangrove forest, South China Sea. BCF = concentration in roots / concentration in sediment. TF = concentration in shoot / concentration in roots.

Species	Cr	Ni	Cu	As	Cd
**BCFs**					
*A*. *marina*	0.36	0.27	0.10	0.04	0.06
*A*. *corniculatum*	0.55	0.34	1.07	0.06	0.09
*S*. *caseolaris*	0.11	0.15	0.30	0.04	0.09
*S*. *apetala*	0.23	0.16	0.23	0.05	0.10
**TFs**					
*A*. *marina*	0.22	0.38	2.72	0.28	0.58
*A*. *corniculatum*	0.21	0.57	0.26	0.24	1.00
*S*. *caseolaris*	0.37	0.98	0.83	0.36	0.57
*S*. *apetala*	0.75	0.57	0.80	0.24	0.50

### 3.3 Heavy metal contamination assessment in sediment

The heavy metals concentrations, background values and marine sediment quality classification in the National Standard of China GB 18668–2002 are summarized for comparison (Table 3). In the present study, the mean Cr, Ni, Cu, As and Cd concentrations were as high as 1.95, 2.03, 7.99, 12.98 and 26.77 times the background values [58]. Class I sediment is suitable for mariculture, nature reserves, endangered species reserves, and leisure activities such as swimming, while Class II can be used for industry and tourism sites and Class III is only suitable for harbors. When compared with the Chinese government’s target values for marine sediment, Cr levels were under Class I standard for Marine Sediment Quality, while, the levels of Cu and Cd were above those of Class I, but lower than those of Class II. The levels of As exceed the Class II standard, but lower than Class III, indicating that As had some influence on the marine sediment quality to a certain extent.

**Table 3:** Heavy metal concentrations in sediment, background level and guideline values of some different criteria (μg g^-1^ dw). Note: TEL, threshold effect level, indicates concentrations below which adverse effects on biota are rarely observed. PEL, probable effects level, indicate concentrations above which adverse effects on biota are frequently observed.

	Cr	Ni	Cu	As	Cd	Reference
Shenzhen Bay	75.74	40.35	79.72	76.60	0.83	This study
Background level	38.80	19.83	9.98	5.90	0.03	[58]
Class Ⅰ	80.00	—	35.00	20.00	0.50	[59]
ClassⅡ	150.00	—	100.00	65.00	1.50	[59]
Class Ⅲ	270.00	—	200.00	93.00	5.00	[59]
Threshold effect level (TEL)	43.40	22.70	31.60	9.79	0.99	[60]
Probable effect level (PEL)	111	48.60	149	33	4.98	[60]

The threshold effect level (TEL) and probable effect level (PEL) for some substances with potential environmental risks were applied to facilitate the interpretation of sediment quality [60, 61]. The TEL represents concentrations below which adverse biological effects rarely occur, while the PEL is defined as the level above which adverse biological effects are expected to occur more often than not [45, 62, 63]. In the present study, mean As concentration was higher than its PEL value (Table 3), indicating that adverse biological effects may occur frequently. Levels of Cr, Ni and Cu were all higher than their TEL values, and below their corresponding PEL benchmarks, suggesting that adverse biological effects caused by Cr, Ni and Cu may be observed occasionally. In addition, Cd level was lower than its TEL and PEL values, indicating no adverse biological effects.

In fact, heavy metals always occur in sediments as complex mixtures. To determine the possible biological effects of combined metals, mean PEL quotients (m-P-Q) for the five heavy metals were calculated using the following formula: m-P-Q = ∑ C_x_/PEL_x_)/n. where C_x_ is the sediment concentration of component x, PEL_x_ is the PEL for compound x and n is the number of components. Three classes of toxicity probability for biota were defined as follows [45, 62]: m-P-Q <0.1 (8% probability of being toxic); 0.11–1.5 (21% probability of being toxic); 1.51–2.3 (49% probability of being toxic); >2.3 (73% probability of being toxic). In the present study, the combination of five studied metals has a 21% probability of being toxic, with the mean PEL quotients as follows: 1.12 (*A*. *marina*), 0.73 (*A*. *corniculatum*), 0.98 (*S*. *caseolaris*), 0.96 (*S*. *apetala*) and 0.74 (Mud flat). Similar results were also observed in intertidal Bohai Bay and the coastal Shandong Peninsula (Yellow Sea), where the mean PEL quotients of studied heavy metals have a 21% probability of being toxic [45, 64, 65].

In the present study, background values were selected as the reference for assessment of heavy metal pollution [36, 37]. The *I*_*geo*_ results showed that the sediments in study area ranged from uncontaminated to heavily contaminated quality for all heavy metals (Table 4). The *I*_*geo*_ values of heavy metals in *A*. *marina* sediment were higher than other sediments,which may be related to land-sourced heavy metal pollutant emission outlets. Another possible explanation may be that the distribution of *A*. *marina* was far away from seawater, and was less affected by tide dilution compared to other mangrove sediments and mud flat. Similar results have also been reported in sediments from mangrove forest and adjacent mud flat in Zhangjiang Estuary [27], where total heavy metal concentrations were forest > forest edge > mudflat. Cu was at moderately contaminated level with uncontaminated to moderately contaminated quality for *A*. *corniculatum* sediment. Except for uncontaminated quality of Cr and Ni in *A*. *corniculatum* sediment, Cr and Ni ranged from uncontaminated to moderately contaminated quality for other sediments. Cd showed moderately to heavily contaminated quality with *I*_*geo*_ ranging from 2.21 to 2.68. Concentrations of As remained at heavily contaminated levels in the study sites, except for moderately to heavily contaminated quality in mud flat. Considering the five metals, the accumulation expressed a descending trend toward sea (except for Cr, Ni and Cu in *A*. *corniculatum* sediment), implying that the location was of great influence in metal accumulation.

**Table 4:** Potential ecological risk assessments of Cr, Ni, Cu, As and Cd in the sediment of Futian mangrove forest, South China Sea.

	Heavy metals							
	Cr	Ni	Cu	As	Cd			
Geo-accumulation index (*I*_*geo*_)								
*A*.*marina*	0.77/UMC	0.76/UMC	1.37/MC	3.68/HC	2.68/MHC			
*A*.*corniculatum*	-0.30/UC	-0.30/UC	0.43/UMC	3.32/HC	2.53/MHC			
*S*. *caseolaris*	0.65/UMC	0.51/UMC	1.20/MC	3.48/HC	2.48/MHC			
*S*. *apetala*	0.73/UMC	0.53/UMC	1.20/MC	3.48/HC	2.48/MHC			
Mud flat	0.55/UMC	0.43/UMC	1.07/MC	2.73/MHC	2.21/MHC			
Potential ecological risk index	$E_{r}^{i}$	$E_{r}^{i}$	$E_{r}^{i}$	$E_{r}^{i}$	$E_{r}^{i}$	RI		
*A*.*marina*	4.63/LR	12.80/LR	48.71/MR	163.42/HR	940.00/VHR	1169.56/VHR		
*A*.*corniculatum*	2.21/LR	6.16/LR	25.30/LR	126.88/CR	850.00/VHR	1010.55/VHR		
*S*. *caseolaris*	4.23/LR	10.76/LR	43.06/MR	141.49/CR	820.00/VHR	1019.54/VHR		
*S*. *apetala*	4.49/LR	10.93/LR	43.27/MR	133.02/CR	860.00/VHR	1051.71/VHR		
Mud flat	3.96/LR	10.22/LR	39.36/LR	84.34/CR	680.00/VHR	817.88/VHR		
Risk assessment code (RAC)								
*A*.*marina*	0.45/NR	8.83/LR	1.86/LR	2.46/LR	12.32/MR			
*A*.*corniculatum*	0.32/NR	6.03/LR	1.95/LR	1.85/LR	8.50/LR			
*S*. *caseolaris*	0.39/NR	7.66/LR	2.63/LR	1.47/LR	15.55/MR			
*S*. *apetala*	0.26/NR	5.44/LR	1.84/LR	1.30/LR	11.35/MR			
Mud flat	0.44/NR	7.95/LR	2.83/LR	1.53/LR	16.08/MR			

Similar to distribution of *I*_*geo*_ values in sediment, the values of each heavy metal $E_{r}^{i}$ in *A*. *marina* sediment were higher than other sediment, indicating higher heavy metal contamination (Table 4). In all sediments, Cr and Ni showed low ecological risk with $E_{r}^{i}$ values lower than 40. Except for low ecological risk of Cu in *A*. *corniculatum* and mud flat, moderate risks of Cu were detected in other sediments. *A*. *marina* sediment demonstrated high risk of As, with considerable risk of As shown in other sediments. Cd showed rather higher $E_{r}^{i}$ values (from 680 to 940), representing very high ecological risk. To quantify the overall potential ecological risks of observed metals, comprehensive potential ecological risk RI is calculated as the sum of all five risk factors. RI represented the sensitivity of the biological community to the toxic metal elements and illustrated the potential ecological risk caused by the overall contamination [66]. In the present study, the RI values in all sediments were higher than 600, indicating very high ecological risk. Furthermore, the *A*. *marina* sediment showed the highest risk compared to other sediments.

### 3.4 Heavy metal speciation in sediment

**Fig 3:**
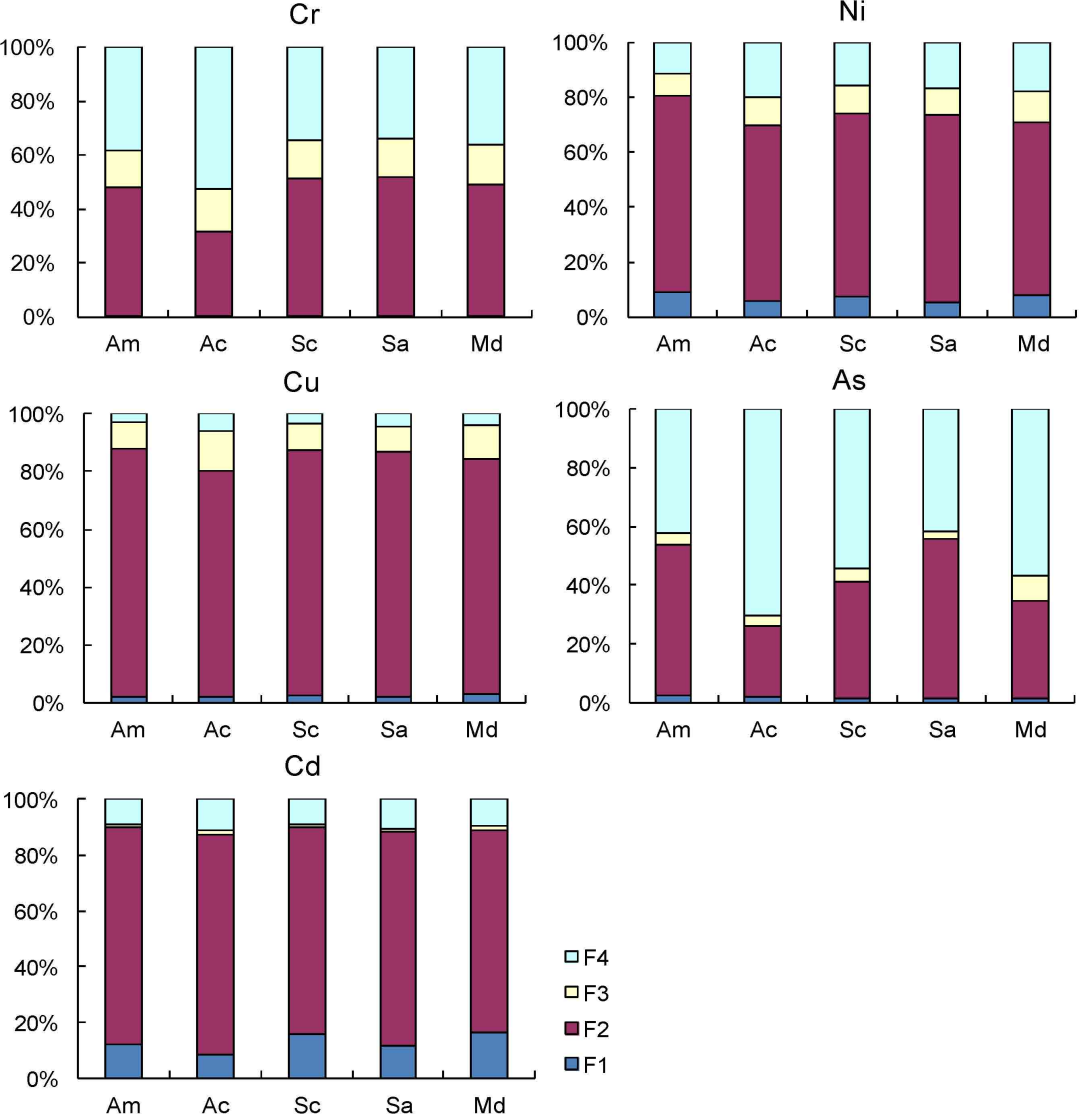
The speciation distributions of heavy metals in sediment in Futian mangrove forest, South China. F1, water/acid-soluble fraction; F2, reducible fraction; F3, oxidizable fraction; F4, residual fraction; Total, total metal concentration. The sample sites from land to sea are: *A*. *marina*—*A*.*corniculatum*—*S*. *caseolaris*—*S*. *apetala*—Mud flat. Am, *Avicennia marina*; Ac, *Aegiceras corniculatum*; Sc, *Sonneratia caseolaris*; Sa, *Sonneratia apetala*; Md, Mud flat.

The toxicity and bioavailability of heavy metals were not only related to their total contents, but also to their speciation [67]. The reducible fraction referred to metal associated with Fe and Mn oxides was reducible, which might be released when subjected to more reducing conditions. The oxidizable fraction referred to metal bound to organic matter and might be released in oxidizing conditions. The water/acid soluble fraction referred to metal that was exchangeable and bioavailable, which was adsorbed on sediment surface by relatively weak electrostatics interactions and sensitive to changes in pH [68, 69]. In the present study, as for Cr, Ni, Cu and Cd, the dominant phase was in the reducible fraction (Fig 3), indicating their strong association with Fe/Mn oxides, from which release of heavy metals into the water column can be expected under prevailing environmental conditions [9, 70]. Beside for reducible fraction, another main phase of As in sediment was residual fractions bound in mineral lattice. The residual fraction of As showed that the release of As is unlikely under environmental conditions, indicating relatively less mobility and bioavailability and less harmful to the environment.

Generally, heavy metals in the water/acid soluble fraction (F1) are bound to carbonates in the weakest strength and could be absorbed by the biota directly [10]. So the percentage of metals in this fraction might indicate the potential risk to the biota. Except for Cu, the RAC values of heavy metals in *A*.*marina* sediment were higher than other sediments (Table 4). Furthermore, the RAC values of Cr in all sediments were lower than one percent, indicating that there was no Cr risk for biota in this mangrove ecosystem. Though As was heavily contaminated according to *I*_*geo*_ and E_r_^i^ criteria based on total concentration, the lower As risk was detected based on water/acid soluble fraction of As. These results indicated that most of As was not biologically toxic. The RAC method has been reported in previous studies [10, 41, 71], and similar different *I*_*geo*_ and RAC results have been reported by Kumar et al. (2012) who found that Mn and Zn had higher risk potential than Fe, Cu, and As based on RAC method; while strongly to moderately contaminated Fe and moderate to uncontaminated other heavy metals were shown by *I*_*geo*_ values. For Ni, Cu and As, whose RAC ranged between one and ten percent, the potential risk remained at low level. Generally, there was medium risk when some metal’s F1 proportion was in a range of 11–30%. In this study, only Cd showed medium risk except for Cd in *A*. *corniculatum* sediment. Compared to previous studies about RAC values of Cd in sewage sludge [2, 13, 72], the RAC levels of Cd were relatively higher, suggesting that heavy Cd contamination has occurred in Shenzhen Bay. Overall, heavy metals introduced by anthropogenic activities has posed a considerable ecological risk to the biota in terms of the speciation (especially for Cd), and deserved enough attention.

## Conclusions

This is the first study to reveal heavy metals in sediment-mangrove plant system in mangrove forest, South China. In the present study, mangrove modified the physicochemical properties of sediments by acidifying soils and increasing organic matter contents. The low BCFs and TFs of heavy metals indicated that mangrove species adopted the exclusion strategy to cope with heavy metal stress. As was toxicologically important due to its concentrations being higher than its TEL and PEL. The sediment had 21% probability of toxicity based on mean PEL quotient. The *I*_*geo*_ values showed that both Cd and As remained at nearly heavily contaminated level in the study sites. $E_{r}^{i}$ and RI results suggested that Cd and As showed rather considerable and very high risk to the surroundings. In terms of the speciation, the majority of each metal element appeared in the reducible fraction. According to the RAC values, heavy metals have posed a considerable ecological risk to the biota, especially for Cd.
